# Paraoxonase-1 Inhibits Oxidized Low-Density Lipoprotein-Induced Metabolic Alterations and Apoptosis in Endothelial Cells: A Nondirected Metabolomic Study

**DOI:** 10.1155/2013/156053

**Published:** 2013-05-22

**Authors:** Anabel García-Heredia, Judit Marsillach, Anna Rull, Iris Triguero, Isabel Fort, Bharti Mackness, Michael Mackness, Diana M. Shih, Jorge Joven, Jordi Camps

**Affiliations:** ^1^Unitat de Recerca Biomèdica, Hospital Universitari de Sant Joan, Institut d'Investigació Sanitària Pere Virgili, Universitat Rovira i Virgili, C. Sant Joan s/n, 43201 Reus, Catalonia, Spain; ^2^Departments of Medicine (Division of Medical Genetics) and Genome Sciences, University of Washington, 3720 15th Avenue NE, Seattle, WA 98195-5065, USA; ^3^Laboratori de Referència Sud, Avenida Cambra de Comerç 42, 43204 Reus, Catalonia, Spain; ^4^Division of Cardiology, University of California, 10833 Le Conte Avenue, Los Angeles, CA 90095-1679, USA

## Abstract

We studied the influence of PON1 on metabolic alterations induced by oxidized LDL when incubated with endothelial cells. HUVEC cells were incubated with native LDL, oxidized LDL, oxidized LDL plus HDL from wild type mice, and oxidized LDL plus HDL from PON1-deficient mice. Results showed alterations in carbohydrate and phospholipid metabolism and increased apoptosis in cells incubated with oxidized LDL. These changes were partially prevented by wild type mouse HDL, but the effects were less effective with HDL from PON1-deficient mice. Our results suggest that PON1 may play a significant role in endothelial cell survival by protecting cells from alterations in the respiratory chain induced by oxidized LDL. These results extend current knowledge on the protective role of HDL and PON1 against oxidation and apoptosis in endothelial cells.

## 1. Introduction

Atherosclerosis, one of the major causes of morbidity and mortality in the Western world, involves complex interactions among endothelial cells of the arterial wall, blood cells, and circulating lipoproteins [1]. Oxidative stress, which is mainly derived from mitochondrial dysfunction, decreases nitrous oxide (NO) synthesis, upregulates the secretion of adhesion molecules and inflammatory cytokines, and is responsible for the oxidation of low-density lipoproteins (LDLs) [2, 3]. These events play a key role in the pathogenesis of atherosclerosis [4, 5].

Paraoxonase-1 (PON1) is an enzyme found in the circulation associated with high-density lipoproteins (HDLs) [6, 7]. The original function attributed to PON1 was that of a lactonase, and lipophylic lactones constitute its primary substrates [8]. PON1 also degrades oxidized phospholipids and, as such, plays a role in an organism's antioxidant system [7]. In the atherosclerosis process, PON1 accumulates in the artery wall [9], and PON1^(−/−)^ mice have been shown to have greater levels of oxidized LDL and larger atheromatous plaques when fed a proatherogenic diet [10]. PON1 also inhibits the production of the proinflammatory chemokine monocyte chemoattractant protein-1 (MCP-1), induced by oxidized LDL in endothelial cells [11]. 

 Despite its potential clinical and biochemical relevance, there is a paucity of studies investigating the influence of PON1 on metabolic alterations when oxidized LDL is incubated with endothelial cells. We reasoned that metabolomics might be a useful tool to evaluate the effects of this enzyme. The study was complemented with an evaluation of oxidative stress and apoptosis in this cell line. 

## 2. Materials and Methods

### 2.1. Experimental Design

We employed primary cultures of human umbilical vein endothelial cells (HUVECs), cultured according to the manufacturer's instructions (Invitrogen, Carlsbad, CA, USA). HUVECs were grown in medium 200 supplemented with low serum growth, 10 mg/L gentamicin and 0.25 mg/L amphotericin (all these reagents were from Invitrogen), and maintained in a humidified incubator at 37°C, with 5% CO_2_. Cells were subcultured when 80%–90% confluent. In all the experiments, cells were plated in 10 cm Petri dishes at a density of 2.5 × 10^3^ cells per dish, and at passage 3. Petri dishes at 70% confluence were incubated over 24 h with isolated human LDL (50 mg/L), oxidized LDL (50 mg/L), oxidized LDL (50 mg/L) + HDL (40 mg/L) from wild type mice, oxidized LDL (50 mg/L) + HDL (40 mg/L) from PON1^(−/−)^ mice, or with serum-free media as controls. All incubations were performed in serum-free media.

Normal human sera were obtained from healthy individuals participating in a population-based study being conducted in our institution. The study was approved by the Ethics Committee (Institutional Review Board) of the Hospital Universitari Sant Joan de Reus. Sera were pooled and used for lipoprotein fractionation and LDL isolation by sequential preparative ultracentrifugation [12, 13]. Human oxidized LDL was prepared by incubation of native LDL with 5 *μ*M CuSO_4_, as described previously [11]. Increased thiobarbituric acid-reactive substances levels were detected in LDL after oxidation (45 versus <0.5 mmol/g protein).

Normal mice were from the C57BL/6J strain (Charles River Labs., Wilmington, MA, USA), and PON1^(−/−)^ mice were the progeny of those provided by the Division of Cardiology of the University of California in Los Angeles and were of a C57BL/6J genetic background [10]. Animals were housed under standard conditions and given a commercial mouse diet (14% Protein Rodent Maintenance Diet, Harlan, Barcelona, Spain) in accordance with our institutional guidelines. At 16 weeks of age, they were sacrificed and approximately 30 mL of sera were pooled for HDL isolation [12, 13]. 

### 2.2. Metabolomics Analyses

The metabolomics platform employed in the present study has been previously described in detail [14]. Briefly, small molecule metabolites from an equivalent amount of cell cytoplasm homogenates were extracted with methanol, and the resulting extract divided into equal fractions for analysis by ultrahigh performance liquid chromatography-tandem mass spectrometry (UPLC-MS/MS; separately under positive mode and negative mode) and gas chromatography-mass spectrometry (GC-MS). Metabolites were identified by comparing the ion data obtained to a reference library of ~2,800 chemical standard entries. Comparisons included retention times, mass (*m*/*z*), and MS or MS/MS spectra. Results of metabolomics measurements are expressed as the mean quotients between the areas under the peak of the different experimental conditions. 

Differences between groups were assessed with Welch's *t*-test for group comparisons. Statistical analyses were performed with the program “R” http://cran.r-project.org/. 

### 2.3. Caspase 9 Western Blot

We analyzed caspase 9 expression in endothelial cell homogenates as a marker of apoptosis pathways. The cytoplasmic homogenates were prepared with a Precellys 24 homogenizer (Bertin Technologies, Montigny-le-Bretonneux, France) [15]. Denaturing electrophoresis was performed in polyacrylamide gels (4–12%) from Invitrogen (Carlsbad, CA, USA). Transfer was performed with the iBlot Gel Transfer Device (Invitrogen). Blotting was performed with the ECL Advanced Western Blotting Detection Kit (GE Healthcare, Fairfield, CT, USA) using a rabbit anticaspase 9 antibody at 1 : 2000 dilution (Abcam, Cambridge, UK) [13].

### 2.4. Measurement of Apoptosis by Flow Cytometry

Cells (300 *μ*L of cell suspension at approximately 10^9^ cells/L) were stained with annexin V conjugated with fluorescein isothiocyanate in the presence of propidium iodide. This enables the detection of phosphatidylserine on the surface of apoptotic cells. We used the Annexin-FITC Kit (Beckman-Coulter, Fullerton, CA, USA) according to the manufacturer's instructions, in a Coulter Epics XL-MLC flow cytometer (Beckman-Coulter).

### 2.5. Measurement of PON1 Activities and Total Peroxide Concentrations

PON1 lactonase activity in the culture's supernatant was measured as the hydrolysis of 5-thiobutyl butyrolactone (TBBL), as described [16]. The assay reagent contained 1 mmol/L CaCl_2_, 0.25 mmol/L TBBL, and 0.5 mmol/L 5,5′-dithio-bis-2-nitrobenzoic acid (DTNB) in 0.05 mmol/L Tris-HCl buffer (pH = 8.0). The change in absorbance was monitored at 412 nm. Activities were expressed as U/L (1 U = 1 mmol of TBBL hydrolyzed per minute). The concentration of total peroxides in the supernatant was determined by a colorimetric enzymatic assay (Immundiagnostik, AG, Bensheim, Germany).

## 3. Results and Discussion

**Figure 1:**
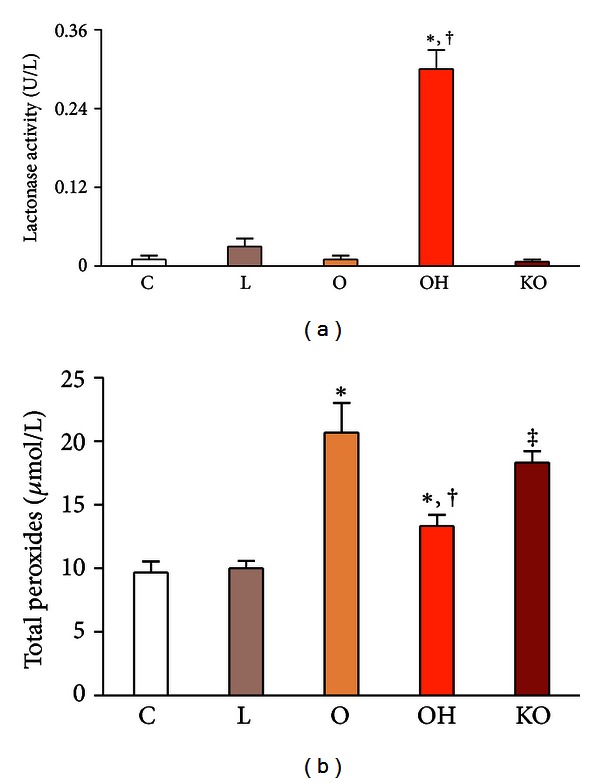
PON1 lactonase activity (a) and total peroxide concentrations (b) in the supernatant of the HUVEC cell culture (*n* = 3, for each experiment). Endothelial cells were incubated over 24 h with 50 mg/L isolated human LDL (L); 50 mg/L oxidized LDL (O); 50 mg/L oxidized LDL + 40 mg/L HDL from wild type mice (OH); 50 mg/L oxidized LDL + 40 mg/L HDL from PON1^(−/−)^ mice (KO); or with serum-free media as controls (C). *P < 0.05, with respect to C; ^†^ *P* < 0.05, with respect to O; ^‡^ *P* < 0.01, with respect to C.

PON1 lactonase activity remained relatively low in supernatants of those cultures not containing added HDL. PON1 lactonase activity significantly increased in those cultures with normal HDL and returned to low levels in those cultures with HDL from PON1^(−/−)^ mice. These results were as expected and provide a quality control of the HDL preparations obtained (Figure 1(a)). Total peroxide concentrations in the supernatants were maximal in the cultures with added oxidized LDL and showed a significant decrease following the addition of normal HDL. This decrease was not as marked following the addition of HDL from PON1^(−/−)^ mice (Figure 1(b)).

We analyzed 124 biochemical compounds by nondirected metabolomics, corresponding to carbohydrate, lipid, amino acid, and nucleotide metabolism, as well as vitamins and xenobiotics. We obtained statistically significant variations in 37 metabolites (Table 1). The main findings corresponded to carbohydrate and phospholipid metabolism and are summarized in the following sections.

### 3.1. Hexose Metabolism

**Figure 2:**
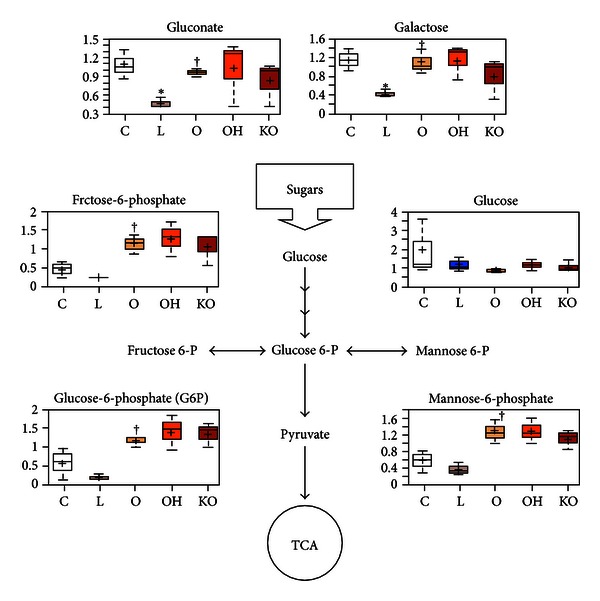
Variations in the hexose metabolites in HUVEC cell homogenates (*n* = 3, for each experiment). Endothelial cells were incubated over 24 h with 50 mg/L isolated human LDL (L); 50 mg/L oxidized LDL (O); 50 mg/L oxidized LDL + 40 mg/L HDL from wild type mice (OH); 50 mg/L oxidized LDL + 40 mg/L HDL from PON1^(−/−)^ mice (KO); or with serum-free media as controls (C). *P < 0.05 with respect to C; ^†^ *P* < 0.05 with respect to L.

The addition of LDL to cultured endothelial cells decreased the levels of gluconate, galactose, and phosphorylated hexose intermediates. These molecules are important entrance intermediates in energy- and biomass-generating pathways such as glycolysis, pentose phosphate, and protein glycosylation. Their decreases suggest that these pathways were activated to a greater extent in endothelial cells treated with LDL, compared to control-treated cells. In contrast, increased levels of gluconate, galactose, and phosphorylated hexose intermediates were seen in all cells that were treated with oxidized LDL, relative to LDL alone, and regardless of whether HDL was also added to the cultures (Figure 2).

### 3.2. Glycolysis and Tricarboxylic Acid (TCA) Cycle

Relative to control cultures, the addition of LDL resulted in increased levels of 3-phosphoglycerate and 2-phosphoglycerate (which are 3-carbon glycolytic intermediates). This same treatment also increased the levels of the TCA cycle intermediates (fumarate and malate) relative to control cultures. An interpretation of these data is that uptake of LDL by endothelial cells results in the generation of acetyl-CoA, which drives flux through the TCA cycle. Increased levels of LDL-generated acetyl-CoA may have relieved the need for carbohydrate-derived precursors, thereby inhibiting glycolytic flux into the TCA cycle and elevating the 3-carbon intermediates. 

By comparison, treatment of endothelial cells with oxidized LDL may have induced levels of oxidative stress that were sufficient to impair normal energy pathways. For example, in cells treated with oxidized LDL, 6-carbon glycolytic intermediates accumulated, whereas the 3-carbon intermediates were reduced. This may be due to changes in glyceraldehyde-3-phosphate dehydrogenase (GADPH; levels or activity) in response to oxidized LDL, since superoxide overproduction inhibits GADPH through a mechanism that involves poly (ADP-ribose) polymerase (PARP) activation [16]. Likewise, TCA cycle intermediates were lower in oxidized LDL-treated cells due, most likely, to the attenuated conversion of 6-carbon glycolytic intermediates to 3-carbon compounds that feed into this cycle through pyruvate and acetyl-CoA. These changes suggest that energy production through glycolysis is impaired, since ATP generation occurs downstream of GADPH activity. 

**Figure 3:**
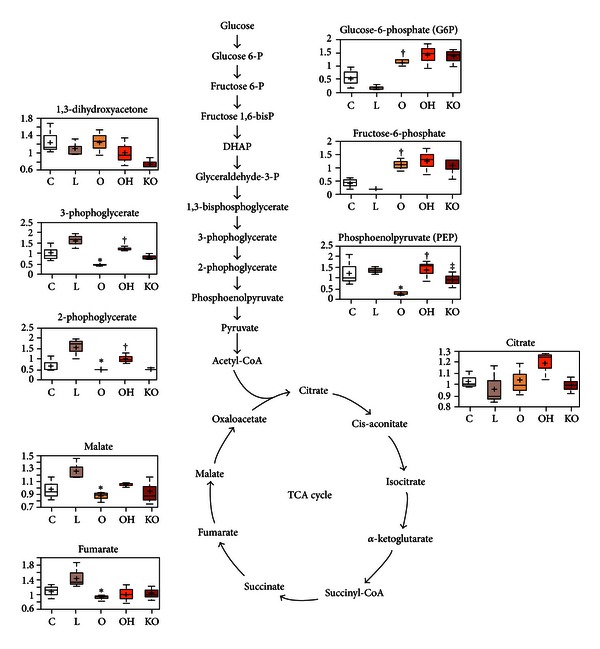
Variations in the metabolites of the glycolytic pathway and tricarboxylic acid cycle in HUVEC cell homogenates (*n* = 3, for each experiment). Endothelial cells were incubated over 24 h with 50 mg/L isolated human LDL (L); 50 mg/L oxidized LDL (O); 50 mg/L oxidized LDL + 40 mg/L HDL from wild type mice (OH); 50 mg/L oxidized LDL + 40 mg/L HDL from PON1^(−/−)^ mice (KO); or with serum-free media as controls (C). *P < 0.05 with respect to L; ^†^ *P* < 0.05 with respect to O; ^‡^ *P* < 0.05 with respect to OH.

The addition of normal HDL to oxidized LDL-treated cells partially reverses its impact on energy metabolism pathways, since levels of the 3-carbon glycolytic intermediates as well as TCA cycle intermediates are more similar to levels observed after LDL treatment alone. It is of note that the impact of addition of HDL from PON1^(−/−)^ mice on these molecules was intermediate between the effects produced by treatment with PON1-containing HDL and of no HDL (Figure 3). This observation is of considerable importance, because PARP activation and its consequent metabolic changes have been associated with endothelial dysfunction in diseases such as atherosclerosis and diabetes [17, 18]. Indeed, the levels of circulating endothelial cells are increased in patients with diabetes mellitus [19], and PON1 has been shown to attenuate diabetes development in mice [20, 21]. Our results suggest that the beneficial role of PON1 may involve, at least in part, a protection against the biochemical changes leading to endothelial dysfunction.

### 3.3. Phospholipid Metabolism

**Figure 4:**
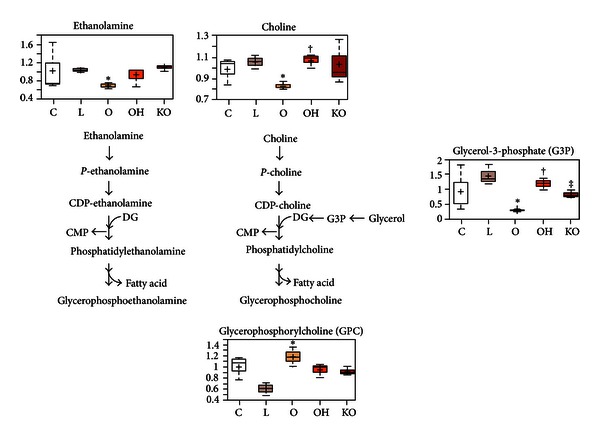
Variations in phospholipid metabolites in HUVEC cell homogenates (*n* = 3, for each experiment). Endothelial cells were incubated over 24 h with 50 mg/L isolated human LDL (L); 50 mg/L oxidized LDL (O); 50 mg/L oxidized LDL + 40 mg/L HDL from wild type mice (OH); 50 mg/L oxidized LDL + 40 mg/L HDL from PON1^(−/−)^ mice (KO); or with serum-free media as controls (C). *P < 0.05 with respect to L; ^†^ *P* < 0.05 with respect to O; ^‡^ *P* < 0.05 with respect to OH.

Levels of choline, ethanolamine, and glycerol-3-phosphate—key building blocks for phospholipids—were similar in endothelial cells following treatment with LDL, when compared to levels in control cells. By comparison, oxidized LDL reduced levels of phospholipid precursors and increased the levels of at least one phospholipid breakdown product. This could indicate that oxidized LDL induces membrane damage, breakdown, or remodeling. As was observed for the energy metabolism pathways, coadministration of normal HDL to oxidized LDL-treated cells reversed, or partially reversed, these deleterious effects. However, the addition of HDL from PON1^(−/−)^ mice only generated subtle changes in phospholipid-related compounds, when compared to treatment with normal HDL (Figure 4). 

### 3.4. Apoptosis

**Figure 5:**
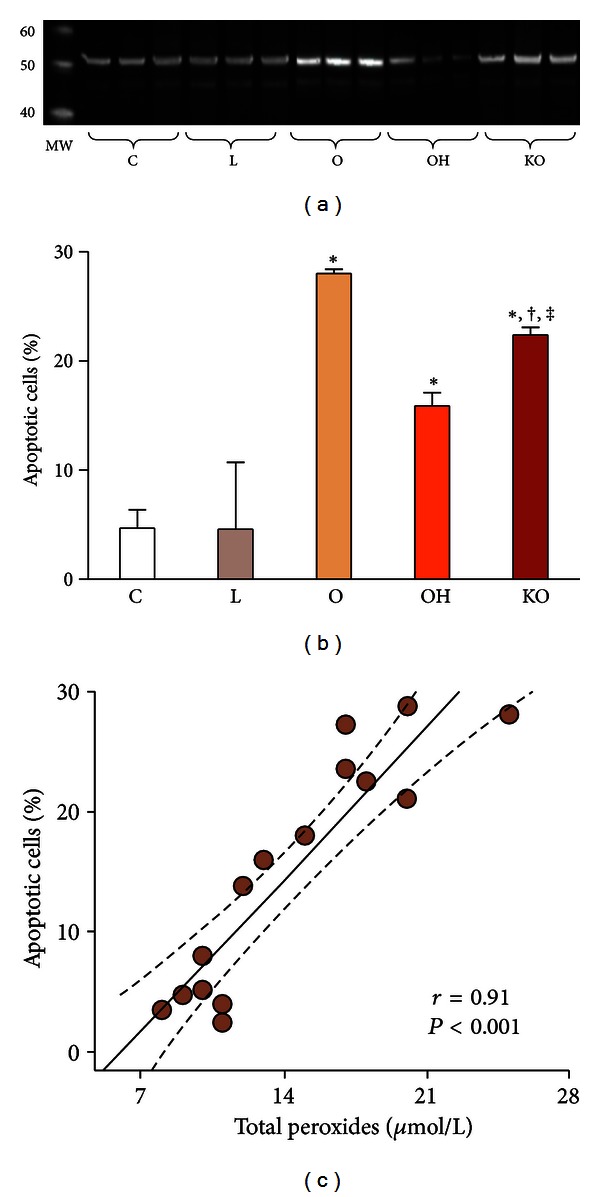
(a) Western blot analyses for caspase 9; (b) percentage of apoptotic cells; (c) relationship between total peroxide concentrations and the percentage of apoptotic cells in HUVEC cell homogenates (*n* = 3, for each experiment). Endothelial cells were incubated over 24 h with 50 mg/L isolated human LDL (L); 50 mg/L oxidized LDL (O); 50 mg/L oxidized LDL + 40 mg/L HDL from wild type mice (OH); 50 mg/L oxidized LDL + 40 mg/L HDL from PON1^(−/−)^ mice (KO); or with serum-free media as controls (C). MW: molecular weight marker. *P < 0.01 with respect to C; ^†^ *P* < 0.05 with respect to OH; ^‡^ *P* < 0.01 with respect to O.

The observation of alterationsin phospholipids levels and the suggested membrane damage channeled us towards investigating the possibility of an increased apoptosis in endothelial cells incubated with oxidized LDL, and a possible protection by introducing HDL as coincubation. Hence, we analyzed caspase 9 protein expression. The activation of this enzyme is a good indicator of apoptosis induction, since caspase 9 plays a determinant role in apoptosome formation [22]. Also, we measured the numbers of apoptotic cells by flow cytometry. We observed that oxidized LDL addition increased caspase 9 expression and the percentage of apoptotic endothelial cells, when compared to control cells and cells treated with normal LDL. Coincubation with normal HDL completely preempted this effect. However, the influence of HDL from PON1^(−/−)^ animals was much lower (Figures 5(a) and 5(b)). We observed a strong direct correlation (*r* = 0.91; *P* < 0.001) between total peroxides concentrations and the percentage of apoptotic cells (Figure 5(c)). Previous studies had shown that increased lipid peroxidation in HDL particles from coronary artery disease patients was associated with an impaired capacity of this particle to stimulate endothelial NO production [23]. Notably, PON1 has been reported to prevent lipid peroxidation in HDL particles and to promote HDL-mediated inactivation of oxidized lipids in LDL. Its activity was shown to be decreased in patients with coronary disease [7]. Further, HDL and PON1 decreased the formation of malondialdehyde-like epitopes and the formation of apoptotic particles in monocytes [24]. A very recent study showed that HDL from healthy people induced the expression of endothelial antiapoptotic protein Bcl-xL and reduced endothelial cell apoptosis *in vitro* as well as *in vivo* in apoE-deficient mice. In contrast, HDL from coronary artery disease patients did not inhibit endothelial apoptosis, failed to activate endothelial Bcl-xL, and stimulated endothelial proapoptotic pathways [25]. Our findings of a decreased oxidized LDL-induced apoptosis by normal HDL, but not by HDL from PON1^(−/−)^ mice, together with a significant association between lipid peroxidation (as measured by total peroxides concentrations) and the percentage of the apoptotic cells would tend to confirm this very recent information. 

 Our results suggest that PON1 may play a significant role in cell survival by improving mitochondrial function. Indeed, mitochondria regulate apoptosis in response to cellular stress signals and, hence, determine whether cells live or die. As such, it is probable that peroxides constitute important candidates in the regulation of cell death, and that mitochondria act as both sensor and effector sites [26]. This could explain the influence of apoptosis-related proteins on mitochondrial respiration. Whether or not this finding has any impact on the atherosclerosis process warrants further exploration. 

## 4. Conclusion

Epidemiological studies have shown that the risk of atherosclerosis is inversely associated with HDL concentrations. The protective effect of this lipoprotein has been attributed, in part, to the antioxidant and anti-inflammatory action of PON1 [27]. We have showed, previously, that PON1 inhibits MCP-1 induction in endothelial cells [11], which suggested a protective role against liver inflammation mediated by MCP-1 [28]. More recent studies indicated that the anti-inflammatory effect of PON1 depends on its association with HDL [29], and that PON1 stimulates HDL antiatherogenicity [30], and macrophage response [31], and increases the duration over which HDL is able to prevent LDL oxidation [32]. 

The present study is novel in using a metabolomic approach to investigate the protective effect of PON1 on endothelial cells incubated with oxidized LDL. We observed important metabolic alterations in human endothelial cells incubated with oxidized LDL. These include an impaired glycolysis, TCA cycle, phospholipids, and activation of apoptotic pathways. These changes were ameliorated by incubation with normal HDL, while HDL isolated from PON1^(−/−)^ mice showed an impaired efficiency to protect against the oxLDL-induced changes. These results extend the current knowledge on the protective role of HDL and PON1 against oxidation in endothelial cells. 

## Figures and Tables

**Table 1:** Heat map of metabolites showing statistically significant differences between groups.

Pathway	Metabolite	L/C*	O/C*	O/L*	OH/O*	KO/O*	KO/OH*
Glycine, serine, and threonine metabolism	Threonine	**1.40**	0.80	*0.57 *	0.96	1.10	1.16
Glutamate metabolism	N-acetylglutamate	1.14	0.84	0.73	1.10	0.92	*0.83 *
Phenylalanine and tyrosine metabolism	Phenylalanine	1.12	**0.63**	*0.56 *	***1.41 ***	**1.19**	0.84
	Tyrosine	1.11	0.62	*0.56 *	**1.57**	1.37	0.87
Valine, leucine, and isoleucine metabolism	Isoleucine	1.36	0.65	*0.48 *	**1.57**	**1.38**	0.88
	Leucine	1.09	**0.70**	*0.64 *	1.25	1.07	0.85
	Valine	1.26	**0.72**	*0.57 *	1.18	1.08	0.91
Urea cycle; arginine-, proline-, metabolism	Praline	1.18	0.89	*0.75 *	0.92	0.97	1.05
Gamma-glutamyl peptides	Gamma-glutamyl-leucine	*0.73 *	0.87	1.19	1.09	1.48	1.36
Amino-sugar metabolism	Fucose	*0.72 *	**0.76**	1.05	1.25	1.06	0.85
Fructose, mannose, galactose, starch, and sucrose metabolism	Galactose	*0.37 *	0.94	***2.58 ***	1.04	0.73	0.7
	Mannose-6-phosphate	0.64	2.21	***3.44 ***	1.01	0.87	0.87
	Glucose-6-phosphate	0.33	2.06	***6.20 ***	1.22	1.17	0.96
	Fructose-6-phosphate	0.50	**2.50**	***5.01 ***	1.13	0.94	0.83
	2-phosphoglycerate	**2.28**	0.67	*0.29 *	***2.17 ***	1.05	*0.48 *
	3-phosphoglycerate	1.62	**0.42**	*0.26 *	***2.92 ***	***1.94 ***	*0.67 *
	1,3-dihydroxyacetone	0.85	0.98	1.15	0.80	*0.60 *	0.75
	Phosphoenolpyruvate	1.06	*0.23 *	*0.22 *	***4.66 ***	***3.05 ***	0.66
Nucleotide sugars, pentose metabolism	Gluconate	*0.43 *	0.89	***2.07 ***	1.06	0.86	0.81
TCA cycle	Fumarate	1.35	0.84	*0.62 *	1.10	1.12	1.02
	Malate	1.31	0.89	*0.68 *	**1.21**	1.07	0.88
Oxidative phosphorylation	Acetyl phosphate	1.00	1.12	1.12	0.84	*0.59 *	0.70
	Phosphate	0.96	1.45	***1.52 ***	0.89	*0.70 *	*0.78 *
Medium chain fatty acid	Laurate (12 : 0)	0.98	1.15	**1.17**	0.92	*0.74 *	*0.81 *
Fatty acid, dicarboxylate	Undecanedioate	1.28	**1.52**	1.19	2.55	*0.70 *	0.28
Glycerolipid metabolism	Ethanolamine	1.00	0.68	*0.68 *	1.28	***1.55 ***	1.21
	Choline	1.07	0.84	*0.78 *	***1.29 ***	1.25	0.96
	Glycerol 3-phosphate	1.54	0.31	*0.20 *	***4.13 ***	***2.87 ***	*0.69 *
	Glycerophosphorylcholine	*0.60 *	1.17	***1.97 ***	0.80	**0.78**	0.97
Purine metabolism, adenine containing	Adenosine 3′-monophosphate	**2.38**	0.73	*0.31 *	***1.70 ***	1.09	*0.64 *
Pyrimidine metabolism, uracil containing	Uracil	1.22	0.48	*0.40 *	***1.94 ***	***1.91 ***	0.99
	Uridine 5′-monophosphate	*0.50 *	1.19	***2.38 ***	0.84	0.92	1.10
Pantothenate and CoA metabolism	Pantothenate	0.98	*0.87 *	0.89	***1.19 ***	1.13	0.95
Riboflavin metabolism	Riboflavin (Vitamin B2)	*0.68 *	0.76	1.11	1.15	1.11	0.97
Benzoate metabolism	4-hydroxy catechol	1.23	1.37	1.11	0.79	*0.43 *	*0.54 *
Chemicals	Glycolate (hydroxyacetate)	1.12	**1.55**	**1.38**	*0.47 *	0.77	**1.64**
	Glycerol 2-phosphate	0.98	0.65	*0.67 *	1.96	1.11	0.57

Endothelial cells were incubated over 24 h with 50 mg/L isolated human LDL (L); 50 mg/L oxidized LDL (O); 50 mg/L oxidized LDL + 40 mg/L HDL from wild type mice (OH); 50 mg/L oxidized LDL + 40 mg/L HDL from PON1^(−/−)^ mice (KO); or with serum-free media as controls (C). Bold italic and italic cells in the Table indicate *P* ≤ 0.05. Bold italic indicates that the mean values are significantly higher; italic indicates significantly lower. Bold text indicates 0.05 < *P* < 0.10. *Results are expressed as the mean quotients of the areas under the peak of the different experimental conditions. For example, galactose values are, on average, 2.58 times higher when endothelial cells are incubated with oxidized LDL than when incubated with native LDL. All measurements were performed in triplicate.

## Abbreviations

DTNB: 5,5′-Dithio-bis-2-nitrobenzoic acid
GADPH: Glyceraldehyde-3-phosphate dehydrogenase
GC-MS: Gas chromatography-mass spectrometry
HDL: High-density lipoproteins
HUVEC: Human umbilical vein endothelial cells
LDL: Low-density lipoproteins
PARP: Poly (ADP-ribose) polymerase
PON1: Paraoxonase-1
TBBL: 5-thiobutyl butyrolactone
UPLC-MS/MS: Ultrahigh performance liquid chromatography-tandem mass spectrometry.
